# Outcome of Antifungal Combination Therapy for Invasive Mold Infections in Hematological Patients is Independent of the Chosen Combination

**DOI:** 10.4084/MJHID.2012.011

**Published:** 2012-02-10

**Authors:** Rafael Rojas, José R. Molina, Isidro Jarque, Carmen Montes, Josefina Serrano, Jaime Sanz, Juan Besalduch, Enric Carreras, José F. Tomas, Luis Madero, Daniel Rubio, Eulogio Conde, Miguel A. Sanz, Antonio Torres

**Affiliations:** 1 The Departments of Hematology and Stem Cell Transplantation Units of University Hospital Reina Sofia, Cordoba. Spain; 2 The Departments of Hematology and Stem Cell Transplantation Units of University Hospital La Fe, Valencia. Spain; 3 The Departments of Hematology and Stem Cell Transplantation Units of University Hospital Marqués de Valdecilla, Santander. Spain; 4 The Departments of Hematology and Stem Cell Transplantation Units of University Hospital Son Dureta, Palma Mallorca. Spain; 5 The Departments of Hematology and Stem Cell Transplantation Units of University Hospital Clinic, Barcelona. Spain; 6 The Departments of Hematology and Stem Cell Transplantation Units of University Hospital MD Anderson, Madrid. Spain; 7 The Departments of Hematology and Stem Cell Transplantation Units of University Hospital Niño Jesús, Madrid. Spain; 8 The Departments of Hematology and Stem Cell Transplantation Units of University Hospital Miguel Servet, Zaragoza. Spain

## Abstract

Invasive mold infection (IMI) remains a major cause of mortality in high-risk hematological patients. The aim of this multicenter retrospective, observational study was to evaluate antifungal combination therapy (ACT) for proven and probable IMI in hematological patients. We analyzed 61 consecutive cases of proven (n=25) and probable (n=36) IMI treated with ACT collected from eight Spanish hospitals from January 2005 to December 2009. Causal pathogens were: *Aspergillus* spp (n=49), *Zygomycetes* (n=6), *Fusarium* spp (n=3), and *Scedosporium* spp (n=3). Patients were classified in three groups according to the antifungal combination employed: Group A, liposomal amphotericin B (L-AmB) plus caspofungin (n=20); Group B, LAmB plus a triazole (n=20), and Group C, voriconazole plus a candin (n=21). ACT was well tolerated with minimal adverse effects. Thirty-eight patients (62%) achieved a favorable response (35 complete). End of treatment and 12-week survival rates were 62% and 57% respectively, without statistical differences among groups. Granulocyte recovery was significantly related to favorable response and survival (p<0.001) in multivariate analysis. Our results suggest that comparable outcomes can be achieved with ACT in high risk hematological patients with proven or probable IMI, whatever the combination of antifungal agents used.

## Introduction

Severe neutropenia and immunosuppression resulting from the use of high-dose chemotherapy in hematological diseases followed, in selected cases, by allogeneic stem cell transplantation (allo-SCT), increase the susceptibility of patients to invasive fungal disease (IFD). The growing use of these aggressive therapies has led to a remarkable increase in the incidence of IFD.^1^–^3^ Although fluconazole prophylaxis has reduced yeast infections in these vulnerable populations, invasive mold infections (IMI), particularly those caused by *Aspergillus* spp, have steadily increased to a 10–20% incidence rate. Furthermore, a high mortality rate for aspergillosis, the most common IMI, has been described, especially among allo-SCT recipients.^1^–^5^ In addition, zygomycosis can reach attributable mortality rates of 91% in these patients.^6^

Many attempts have been made to decrease the incidence and mortality of IFD in patients with hematological malignancies. Strategies including empiric therapy,^7^–^9^ pre-emptive treatment^10^–^13^ and improved prophylactic regimens with new azoles have been proposed.^14^–^19^ Monotherapy with new antifungal agents such as candins, triazoles and liposomal amphotericin B (L-AmB) has also been used as treatment for proven or probable IFD.^20^–^23^ However, though improved survival rates have been documented with the use of these individual agents, overall response and treatment outcome remain suboptimal in cases of IMI with high mortality rates.

Recently, several retrospective or uncontrolled clinical reports^24^–^30^ and, as far as we know, only one prospective randomized study^31^ have addressed the potential benefit of using combinations of new antifungal agents in the treatment of IFD. This issue is conceptually promising, because an additive activity or even synergy of antifungal drugs might be expected.^32^ Although these studies provide evidence supporting the use of this approach, the advantages of combined therapy have not yet been clearly demonstrated.^25^–^32^ Therefore, it is still unclear if antifungal combined therapy (ACT) is superior to monotherapy in severely neutropenic and/or immunocompromised patients with life-threatening IFD.

In this retrospective, observational, multicenter study, we describe the results of ACT in 61 proven or probable cases of IMI occurring in high-risk hematological patients treated with intensive chemotherapy or allo-SCT.

## Patients and Methods

We have retrospectively analyzed all consecutive cases of high-risk hematological patients with proven or probable IMI treated with ACT in eight tertiary university hospitals in Spain over a 5-year period (January 2005 – December 2009). For inclusion in the analysis, patients must have received at least seven days of ACT. Antifungal agents used as primary prophylaxis, empiric treatments or in ACT depended on the specific policies of participating hospitals.

## Definitions

Diagnosis of IMI was established according to the revised EORTC/MSG criteria.^33^ Responses to antifungal therapy were defined according to the MSG/EORTC consensus criteria^34^ as either favorable response (complete or partial) or failure (stable disease, progression or death from any cause).

Prophylaxis was defined as primary when patients did not have a previous history of IFD, while it was defined as secondary in patients with a previous IFD history but no signs or symptoms of fungal infection when the new hematological treatment was initiated.

ACT was started when the diagnosis of proven or probable IFD was made. The following doses were used in the treatment of adult patients: voriconazole, loading dose of 6 mg/kg/12 hours × 2 doses followed by 4 mg/kg/12 hours; posaconazole, 400 mg/12 hours; caspofungin, 70 mg on day 1 and 50 mg/day starting from day 2; anidulafungin, 200 mg on day 1 and 100 mg/day after and L-AmB, 3 mg/kg/day. The doses used in the treatment of children were: voriconazole, 7 mg/kg/12 hours; caspofungin, 70 mg/m^2^ (maximum 70 mg) on the first day and 50 mg/m^2^ (maximum 50 mg) each subsequent day and L-AmB, 3 mg/kg/day. All drugs were given intravenously but posaconazole was administered orally. Azole plasma levels were not monitored.

*De novo* ACT was defined as the combination of two antifungal drugs not used before for prophylaxis or empiric treatment. Sequential ACT occurred when an antifungal drug was added to another already being used for prophylaxis or empiric treatment.

## Statistical Analysis

Data were collected in a SPSS database and all statistical results were performed using SPSS version 17. Either the chi-square or the Fisher’s exact test were used to compare data. Overall survival was analyzed using temporal series Kaplan-Meier analysis and comparisons between different treatments groups were performed using the log-rank test. For multivariate analysis, logistic regression was used. A p value of less than 0.05 was defined as statistically significant.

## Results

### Patients

Sixty-one patients were included in the study. The mean age was 43 years (range, 3–73) with 7 patients younger than 18 years. Thirty-four patients had received intensive chemotherapy for induction (n=28) or consolidation (n=6). Twenty-five patients were recipients of allo-SCT and one patient received an autologous SCT. The main demographic and clinical data are shown in Table 1.

Fifty-four patients (89%) were severely neutropenic (absolute neutrophil count <500/μL for at least 10 consecutive days) at the onset of IFD; of these, 9 had at the same time acute graft versus host disease (GvHD). Only 7 patients developed an IFD without severe neutropenia: 3 patients had acute GvHD, 3 patients chronic GvHD and 1 patient severe aplastic anemia under immunosuppressive treatment.

Prior antifungal prophylaxis was administered to 60 patients (23 voriconazole, 18 fluconazole, 18 itraconazole and 1 L-AmB). In 35 patients, prophylaxis was stopped when empirical antifungal treatment was initiated (9 patients were given voriconazole, 16 caspofungin and 10 L-AmB). The patient not receiving prophylaxis started empirical treatment with voriconazole. Only 3 patients with prior history of IFD (2 candidemias and 1 probable pulmonary aspergillosis) received secondary prophylaxis (1 fluconazole, 2 voriconazole).

*De novo* ACT was started in 26 patients. In 23 other patients, combined treatment was initiated by adding a new antifungal agent to the empirical treatment. In the remaining 12 patients, a second antifungal drug was added to the prophylaxis regimen.

The diagnosis of proven IFD was confirmed in 25 patients, while the remaining 36 patients met the criteria for probable IFD. The molds isolated in proven IFD cases were as follows: *Aspergillus fumigatus* (n=6), *A. flavus* (n=3), *A. terreus* (n=1), *Aspergillus* spp. (n=3), *Mucor* spp. (n=6), *Fusarium oxysporum* (n=1), *Fusarium* spp. (n=2), *Scedosporium apiospermum* (n=2) and *S. prolificans* (n=1). All cases of probable IFD were aspergillosis. All patients with mucormycosis were treated with ACT and surgery, with the exception of one patient with pulmonary mucormycosis diagnosed at postmortem examination.

### Antifungal Combination Therapy

The mean duration of ACT was 31 days (range, 8–127). Three antifungal combination groups were identified: group A, L-AmB plus caspofungin (20 patients); group B, L-AmB plus triazole (voriconazole 16 and posaconazole 4) and group C, voriconazole plus candin (20 caspofungin and 1 anidulafungin).

### Toxicity

ACT was well tolerated except for a mild increase in liver enzymes in patients receiving voriconazole and a mild increase in serum creatinine levels in two patients receiving L-AmB. In one case, LAmB treatment was discontinued for two days returning the serum creatinine level to normal following a dose reduction from 3 to 1.5 mg/kg/day for two additional days. Hypokalemia was observed in patients receiving L-AmB, but they responded well to potassium supplements.

### Outcome

Thirty-eight patients had a favorable response to ACT [35 complete response (CR) and 3 partial responses (PR)]. The survival rate at the end of treatment was 62% for the whole series and the 12-week survival rate after initiation of ACT was 57%.

In group A, 13 (65%) patients had favorable response (11 CR, 2 PR), in group B, 12 (60%) patients (11 CR, 1 PR response) and in group C, 13 (62%) patients (13 CR). We found no statistical differences in terms of clinically favorable responses among groups A, B and C (Table 2).

**Figure 1:**
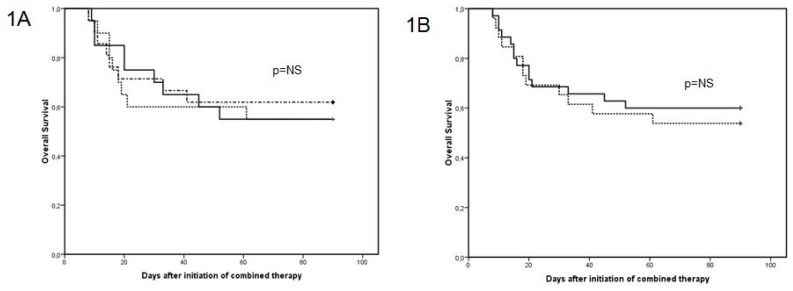
**A.** Kaplan-Meier curves comparing the overall survival at 12 weeks related to different ACT in the global series: Group A: L-AmB + candin (——), Group B: L-AmB + triazole (-----) and Group C: triazole + candin (- - - -). **B.** Kaplan-Meier curves comparing the overall survival at 12 weeks related to different antifungal combinations: “*de novo*” ACT (-----) and ACT as the result of adding an antifungal drug to a treatment already being used for prophylaxis or empiric treatment (——) in the global series.

Survival rates at the end of treatment for groups A, B and C were 65%, 60% and 62%, respectively. The probability of survival at 12 weeks for groups A, B, and C were 55%, 55% and 62%, respectively (Figure 1A). We found no significant differences in the probability of survival at 12 weeks between patients treated with *de novo* ACT compared with those treated with sequential ACT through addition of another antifungal agent to previous prophylaxis or empiric therapy (Figure 1B).

A total of 20 out of 27 patients (74%) receiving induction or consolidation chemotherapy for AML achieved a favorable response to ACT while a favorable response occurred in only 14 out of 25 recipients of allo-SCT (56%). Survival at 12 weeks was higher in AML (64%) compared to allo-SCT (52%). However these differences were not statistically significant.

Twenty-three patients died. Deaths were related to IFD alone (n=8), IFD in the setting of progressive underlying disease (n=14), and cytomegalovirus infection plus acute GvHD (n=1). From the end of the combined treatment to 12 weeks after the initiation of ACT, 3 additional patients died due to chronic GvHD (n=1) and leukemia relapse (n=2). IFD-related mortality at 12 weeks was 39%.

### Antifungal Combination Therapy in Aspergillosis

Invasive aspergillosis (IA) was diagnosed in 49 patients (13 proven and 36 probable). A total of 32 (65%) patients responded favorably to treatment (30 CR and 2 PR), and the end of treatment and 12-week survival rates were 65% and 61%, respectively.

**Figure 2:**
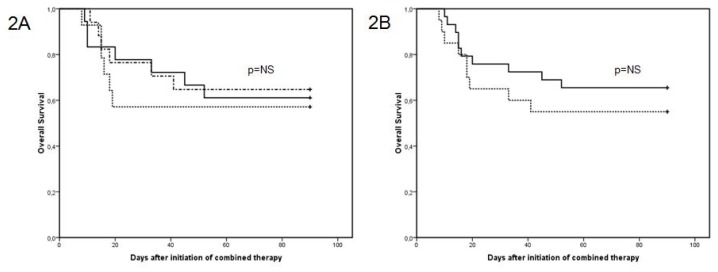
**A.** Kaplan-Meier curves comparing the overall survival at 12 weeks with respect to different ACT in patients with IA: Group A: LAmB + candin (——), Group B: L-AmB + triazole (-----) and Group C: triazole + candins (- - - -). **B.** Kaplan-Meier curves comparing the overall survival at 12 weeks with respect to different antifungal combinations: “de novo” ACT (-----) and ACT as the result of adding an antifungal drug to the used for prophylaxis or empiric treatment (——) in patients diagnosed of IA.

In group A, 13 of 18 patients (72%) responded (11 CR and 2 PR), in group B, 8 of 14 patients (57%, 8 CR) and in group C, 11 of 17 patients (65%, 11 CR). We found no statistical difference between response rates among groups A, B and C (Table 3). Furthermore, there were no significant differences in survival at 12 weeks among groups A, B and C (Figure 2A). Moreover, no significant differences were observed between patients treated with *de novo* ACT and those with sequential ACT (Figure 2B). The probability of death at 12 weeks attributable to IA was found to be 34%.

### Analysis of Prognostic Factors

Univariate analysis revealed that the use of non-active mold prophylaxis (fluconazole or no prophylaxis) influenced unfavorably the ACT response (p=0.03) and mortality (p=0.03), while granulocyte recovery and complete remission of the underlying malignancy improved the response to ACT (p<0.001 and p=0.009, respectively) and decreased mortality (p<0.001 and p=0.002 respectively). Other clinical variables such as age, stem cell transplantation, *de novo* ACT and IA did not show significant influence on response or mortality. Logistic regression multivariate analysis revealed that granulocytic recovery was the only statistically significant variable (p<0.001) (Table 4).

When we analyzed these variables separately in groups A, B and C only granulocyte recovery was statistically significant for response (p=0.004 and p=0.02 for group A and B, respectively) and survival at 12 weeks (p=0.02 and p=0.007 for group A and B, respectively). In group C all patients with no granulocyte recovery died.

## Discussion

This study shows that ACT seems to be suitable for the treatment of IFD in severely immunocompromised patients.^20^–^23^ Our results are in line with those previously reported.^24^–^31^ It should be noted that the three types of ACT employed in our patients resulted in similar response rates in both the overall series and in patients with IA. These findings do not support the results that in both *in vitro* and animal models have been reported.^35^,^36^ These studies have suggested an antagonism with the combination of L-AmB and voriconazole. Probably, as Segal & Steinbach^37^ have stated, a major limitation in the antifungal field is the lack of consistent ability to use *in vitro* models to predict clinical efficacy. In general, antagonism has not been apparent in the clinical setting. In fact, a recent study by Cornely *et al*^38^ suggested that prior azole prophylaxis or therapy did not affect overall response nor mortality in patients who were treated with L-AmB for IMI (49% response rate in patients given previously azoles *vs.* 46% in those without prior azole exposure). Survival at 12 weeks was also similar (64% *vs.* 66%).

Although our study is not a prospectively randomized trial, we believe it provides valuable information on the potential impact of ACT in seriously ill patients with hematological diseases. In fact, as far as we know, the only prospective randomized trial reported is a pilot study by Caillot et al.^31^ which included a small number of patients (15 patients in each arm). High dose L-AmB (10 mg/kg/day) was compared with a combination of standard dose L-AmB (3 mg/kg/day) plus caspofungin in 30 patients with hematologic malignancies and proven or probable invasive aspergillosis (COMBISTRAT trial). Response rates at the end of treatment were 67% for the combination *vs.* 27% with monotherapy (p= 0.03). Survival rates at 12 weeks were similar, 100% for the combination and 80% for monotherapy. Nevertheless, this study indicated the superiority of standard L-AmB plus caspofungin over high-dose L-AmB monotherapy, which in addition had a higher toxicity. On the other hand, the consistently better results in most retrospective case studies using ACT in the treatment of probable and proven IFD are similar to those obtained in the present study.^24^–^31^

An increasing number of patients with IFD and life-threatening conditions can be cured with ACT when the underlying disease is under control. It should be noted that, in our case series, as in other published reports,^39^ ACT was well tolerated with only minor toxicity. In contrast, some case series in which single drugs were used had similar or higher toxicity than those reported in the present study.^20^,^22^ However, retrospective studies usually suffer from potential bias. For instance, in our study only patients who survived at least 7 days from the start of ACT were considered for analysis, and consequently, patients with very severe infections may have been excluded from the study. This criticism may be applied to most clinical trials that exclude patients with poor performance status (ECOG ≥2), life-expectancy less than one week or severe organ dysfunction.

Regarding prognostic factors, granulocyte recovery resulting from the control of the underlying disease is the most important variable in IFD patients regardless of the ACT used. The use of ACT instead of monotherapy may more efficiently stabilize the IFD, preventing fatal progression while neutrophil counts recover and the immune system is restored. This finding raises again the question about the potential utility of granulocyte transfusions as adjunctive treatment. After years of controversy, the RING (Resolving Infections in Neutropenia with Granulocytes) study has been designed to evaluate the effectiveness of transfusing large numbers of G-CSF/dexamethasone mobilized granulocytes from community donors. When available, the results of this multicenter phase III randomized controlled clinical trial may be of great value to definitely address this unresolved issue.^40^

## Figures and Tables

**Table 1:** Patient characteristics

Characteristics	All patients n (%)=61 (100)	Group A n (%)=20 (33)	Group B n (%)=20 (33)	Group C n (%)=21 (34)
**Age mean (range)**	43 (3–73)	44 (8–64)	48 (21–73)	39 (3–72)
**Sex (M/F)**	34 (56)/27 (44)	11 (55)/9 (45)	14 (70)/6 (30)	9 (43)/12 (57)
**Underlying disease**				
AML	27 (44)	11 (55)	7 (35)	9 (43)
ALL	19 (31)	3 (15)	9 (45)	7 (33)
SAA	2 (3)	1 (5)	1 (5)	0
MDS	8 (13)	3 (15)	2 (10)	3 (14)
NHL	3 (5)	1 (5)	1 (5)	1 (5)
MM	1 (2)	1 (5)	0	0
BPL	1 (2)	0	0	1 (5)
**Allo-SCT**	25 (41)	8 (40)	8 (40)	9 (43)
HLA-identical sibling	10 (16)	2 (10)	2 (10)	6 (29)
Adult unrelated donor	4 (6)	1 (5)	2 (10)	1 (5)
Cord blood	11 (18)	5 (25)	4 (20)	2 (10)
**High risk condition**				
SN	45 (74)	17 (85)	13 (65)	15 (71)
Acute GvHD + SN	9 (15)	1 (5)	3 (15)	5 (24)
Acute GvHD	3 (5)	1 (5)	2 (10)	0
Chronic GvHD	3 (5)	1 (5)	1 (5)	1 (5)
Severe IS	1 (2)	0	1 (5)	0
**Acute GvHD**				
Grade 0	13 (21)	6 (30)	3 (15)	4 (19)
Grade I–II	9 (15)	2 (10)	3 (15)	4 (19)
Grade III–IV	3 (5)	0	2 (10)	1 (5)
***IFD diagnosis (EORTC/MSG)***				
Proven	25 (41)	8 (40)	10 (50)	7 (33)
Probable	36 (59)	12 (60)	10 (50)	14 (67)
**Sites of Infection**				
Pulmonary	51 (84)	19 (95)	13 (65)	19 (90)
Disseminated	8 (13)	1 (5)	5 (25)	2 (10)
Paranasal sinuses	2 (3)	0	2 (10)	0
***Fungal Pathogen***				
*Aspergillus* spp	49 (67)	18 (90)	14 (70)	17 (81)
*Zygomycetes*	6 (10)	0	3 (15)	3 (14)
*Scedosporium* spp	3 (5)	1 (5)	1 (5)	1 (5)
*Fusarium* spp	3 (5)	1 (5)	2 (10)	0

M: male, F: female, HLA: human leukocyte antigen. AML: acute myeloid leukemia. ALL: acute lymphoblastic leukemia. SAA: severe aplastic anemia. MDS: myelodysplastic syndrome. NHL: non-Hodgkin lymphoma. MM: multiple myeloma. BPL: byphenotypic leukemia. SN: severe neutropenia. GvHD: Graft versus Host Disease. **Group A:** L-amB plus caspofungin. **Group B:** L-amB plus triazole (16 voriconazole and 4 posaconazole). **Group C:** Voriconazole plus candin (20 caspofungin and 1 anidulafungin)

**Table 2:** Therapeutic results of different ACT regimens in the global series (n=61)

	Group A n=20 n (%)	Group B n=20 n (%)	Group C n=21 n (%)
**Response**			
***Favorable***	13 (65)	12 (60)	13 (62)
Complete	11 (55)	11 (55)	13 (62)
Partial	2 (10)	1 (5)	0 (0)
***Failure***	7 (35)	8 (40)	8 (38)
**Death related to IFD**	6 (30)	8 (40)	8 (38)
**Alive at the end of treatment**	13 (65)	12 (60)	13 (62)
**Alive at 12 weeks**	11 (55)	11 (55)	13 (62)

IFD indicates invasive fungal disease. Group A: L-amB plus caspofungin. Group B: L-amB plus triazole (16 voriconazole and 4 posaconazole). Group C: Voriconazole plus candin (20 caspofungin, 1 anidulafungin).

**Table 3:** Therapeutic results of different ACT regimens in patients diagnosed with proven or probable invasive aspergillosis (n=49)

	Group A n=18 n (%)	Group B n=14 n (%)	Group C n=17 n (%)
**Response**			
*Favorable*	13 (72)	8 (57)	11 (65)
Complete	11 (61)	8 (57)	11 (65)
Partial	2 (11)	0 (0)	0 (0)
***Failure***	5 (28)	6 (43)	6 (35)
**Death related to IFD**	5 (28)	5 (36)	6 (35)
**Alive at the end of treatment**	13 (72)	8 (57)	11 (65)
**Alive at 12 weeks**	11 (61)	8 (57)	11 (65)

IFD indicates invasive fungal disease. Group A: L-amB plus caspofungin. Group B: L-amB plus triazole (12 voriconazole and 2 posaconazole). Group C: Voriconazole plus candin (16 caspofungin, 1 anidulafungin)

**Table 4:** Univariate analysis of factors influencing response to ACT and 12-week overall survival

		Response		Survival at 12 weeks	
*Variable*	*n (%)*	n (%)	p value	n (%)	p value
**Age (adults/children)**	54 (89)/7 (12)	35 (65)/3 (43)	0.3	32 (59)/3 (43)	0.4
**Stem cell transplantation (yes/no)**	26 (43)/35 (57)	15 (58)/23 (66)	0.5	14 (54)/21 (60)	0.6
**Non-active mold prophylaxis (yes/no)**	19 (31)/42 (69)	8 (42)/30 (71)	**0.03**	7 (37)/28 (67)	**0.033**
**ACT “de novo” (yes/no)**	26 (43)/35 (57)	15 (58)/23 (66)	0.5	14 (54)/21 (60)	0.6
Granulocytic recovery* **(yes/no)**	35 (57)/26 (43)	33 (94)/5 (19)	**<0.001**	31 (89)/4 (15)	**<0.001**
**Aspergillosis IFD (yes/no)**	49 (80)/12 (20)	32 (65)/6 (50)	0.3	30 (61)/5 (42)	0.2
**Complete remission status (yes/no)**	44 (72)/17 (28)	32 (73)/6 (35)	**0.009**	31 (71)/4 (24)	**0.002**

ACT indicates antifungal combination therapy. IFD: invasive fungal disease.

* Only granulocyte recovery was significantly associated to an improved response and survival in multivariate analysis (p<0.001).
